# Insights into Clinical Features and Outcomes of Adrenal Cortical Carcinosarcoma

**DOI:** 10.3390/diagnostics12102419

**Published:** 2022-10-06

**Authors:** Zachery Branham, Ashley D. Fox, Asad Ullah, Nikhil G. Patel, Martha Terris, Jigarkumar Parikh

**Affiliations:** 1 Department of Internal Medicine, Medical College of Georgia at Augusta University, Augusta, GA 30912, USA; 2 Department of Pathology, Vanderbilt University Medical Center, Nashville, TN 37232, USA; 3 Department of Pathology, Medical College of Georgia at Augusta University, Augusta, GA 30912, USA; 4 Department of Urology, Medical College of Georgia at Augusta University, Augusta, GA 30912, USA; 5 Department of Hematology/Oncology, Georgia Cancer Center, Augusta, GA 30912, USA

**Keywords:** adrenal carcinosarcoma, carcinosarcoma, adrenal cancer

## Abstract

Adrenal cortical carcinosarcomas are a rare and typically aggressive malignancy with few reported cases in medical literature. We present a case of a 78-year-old female who presented with complaints of fatigue and right shoulder pain. Imaging of the abdomen with computed tomography visualized a large mass in the right upper quadrant. The mass was radiologically described as a 22 × 17 × 13 cm heterogeneous mass with its epicenter in the area of the right adrenal gland, with medial and peripheral effacement of all structures in the right upper quadrant. Non-contrasted images demonstrated anterior mid-portion calcifications. The mass parasitized its blood supply from several surrounding structures, including the liver and right psoas muscle, and extensively invaded the psoas muscle. Resection of the mass was performed with pathology, which revealed a high mitotic index and nuclear atypia with two morphologically and immunophenotypically distinct components. One of these components stained positively for calretinin and inhibin, which is indicative of adrenal cortical carcinoma; the other exhibited strong expression of vimentin and desmin, which was concordant with sarcomatous change and confirmed the diagnosis of adrenal cortical carcinosarcoma. This unique histology with both carcinomatous and sarcomatous components presents a diagnostic challenge for clinicians. As such, adrenal carcinosarcomas should be kept on the differential when evaluating retroperitoneal masses. Additionally, this study includes a review of 34 previously reported cases of adrenal cortical carcinosarcomas along with a discussion about the future exploration of this pathology.

## 1. Introduction

Adrenal cortical carcinosarcoma is an exceedingly rare malignancy of the adrenal gland. This pathology is so uncommon that knowledge and statistical evidence concerning it relies on case reports. It is the least common variant of adrenal cortical carcinoma [1]. Okazumi et al., described the first known case in 1987 [2]. This malignancy is notable for exhibiting both carcinomatous features and mesenchymal differentiation [3]. Prognosis is usually very poor [4]. Treatment begins with surgical resection; however, disease recurrence is common [1]. We present a case detailing a 78-year-old female with confirmed adrenal cortical carcinosarcoma to further progress medical knowledge concerning this rare pathology. We also performed a literature review of published cases to summarize current knowledge and to create discussion on the assessment and treatment of adrenal cortical carcinosarcomas.

## 2. Case Presentation

**Figure 1:**
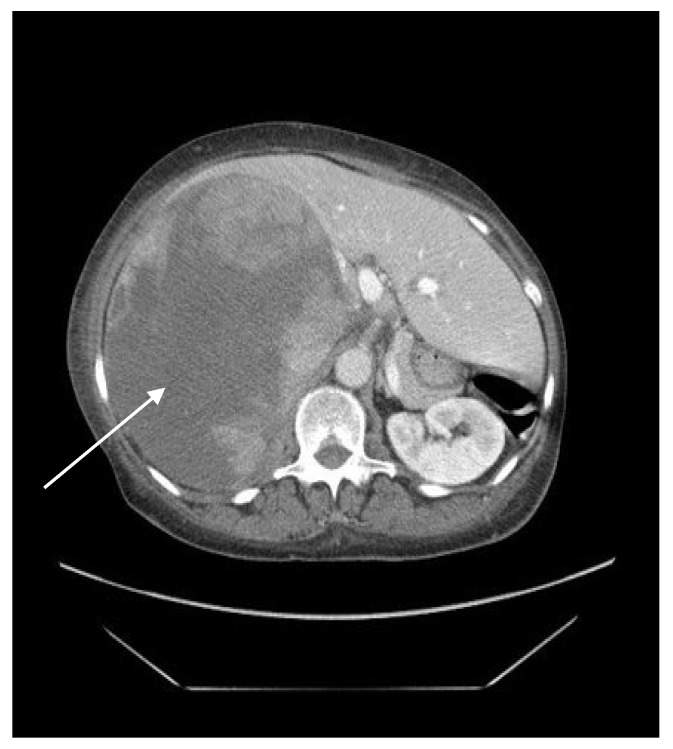
Contrast-enhanced CT scan of the abdomen and pelvis, axial view. Large heterogeneous mass visualized (arrow).

A 78-year-old African American female with a past medical history of long-standing hypertension and diabetes mellitus, coronary artery disease, and tobacco use presented with complaints of fatigue and right shoulder pain for two weeks. The patient also noted a palpable mass in her right upper quadrant that she had noticed two months previously. On arrival to the hospital, her vital signs were significant with a blood pressure of 175/90 mmHg, but was otherwise within normal limits. The patient’s white blood cell count was elevated to 23 thousand cells per cubic millimeter and alkaline phosphatase was elevated to 227 units per liter. The physical exam noted a palpable mass in the patient’s right upper quadrant. There were no obvious signs of hormone or endocrine dysfunction. The patient’s hypertension and diabetes mellitus had been present for many years. A contrast-enhanced computed tomography scan of the abdomen and pelvis was obtained and demonstrated a heterogeneous mass measuring 22 × 17 × 13 cm with its epicenter in the area of the right adrenal gland (Figure 1). Effacement was noted in the structures in the right upper quadrant, as well as in the right kidney and renal vein. Blood supply to the mass was parasitized from the right lobe of the liver, right inferior phrenic artery, inferior adrenal artery, and the capsular branches off the right kidney. The mass additionally invaded the right psoas muscle, extending from the level of the diaphragm to at least the level of the L3 vertebra. A diffuse soft tissue edema, likely the result of vena cava compression, was additionally noted. The left adrenal gland appeared normal. Serum tumor markers were checked. The patient was noted to have an elevated cancer antigen 125 (CA-125) at 62 U/mL (normal range 0.00–30.20 U/mL). Alpha-fetoprotein (AFP), carcinoembryonic antigen (CEA), and carbohydrate antigen 19-9 (CA 19-9) levels were within normal limits. Adrenocortical hormone levels and metanephrine assays were not assessed in this patient.

**Figure 2:**
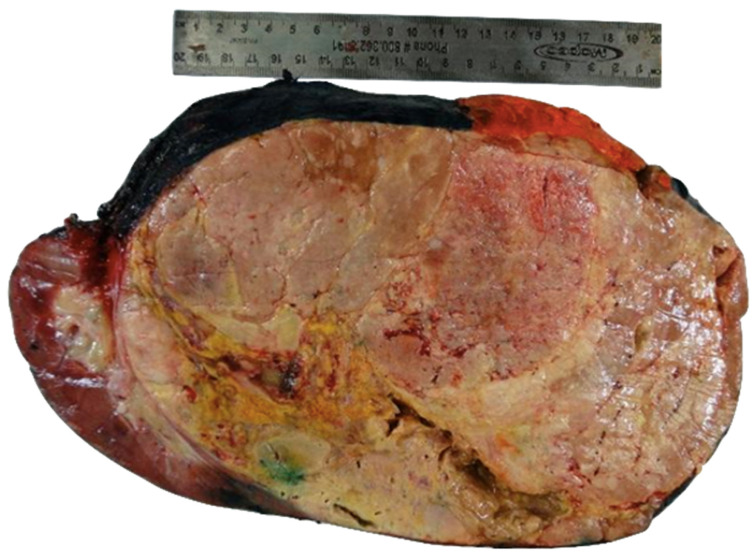
Gross image of tan-yellow mass with areas of hemorrhage and necrosis.

The patient was taken to the operating room for resection of the mass. Grossly, the mass measured 27 × 17 × 12 cm and weighed 3.3 kg. The serial section of the mass revealed a tan-yellow hemorrhagic and necrotic cut surface with an area of capsular disruption (Figure 2). The normal adrenal gland was not identified.

**Figure 3:**
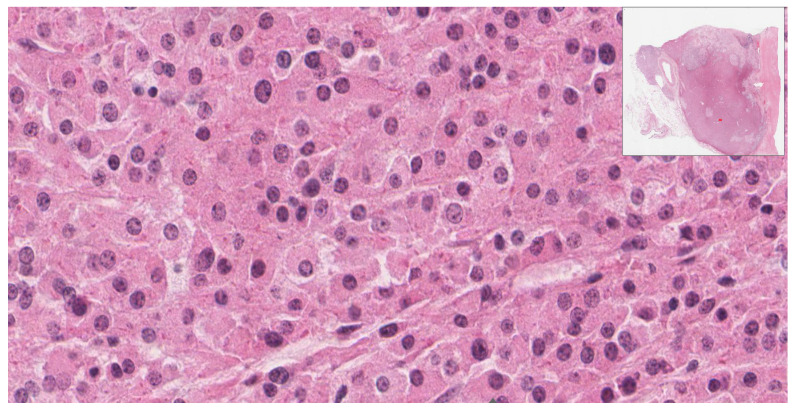
(40×): Carcinoma component: epithelioid cells with abundant cytoplasm and round nuclei.

**Figure 4:**
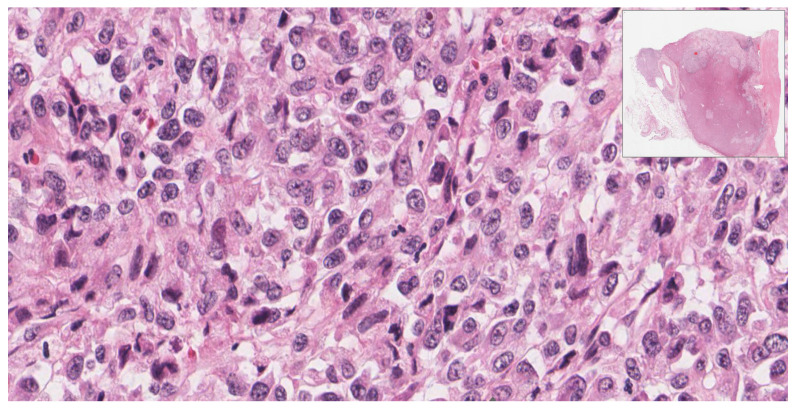
(40×): Sarcoma component: pleomorphic spindle cells with nuclear hyperchromasia.

Histologically, the lesion revealed solid sheets of the tumor with heterogeneous components and necrosis. The carcinoma component of the tumor consisted of polygonal cells with abundant eosinophilic granular cytoplasm and round nuclei with an open chromatin pattern (Figure 3). The sarcoma component was comprised of pleomorphic spindle cells, eosinophilic cytoplasm, identifiable mitosis, and dark, pleomorphic, hyperchromatic nuclei (Figure 4).

**Figure 5:**
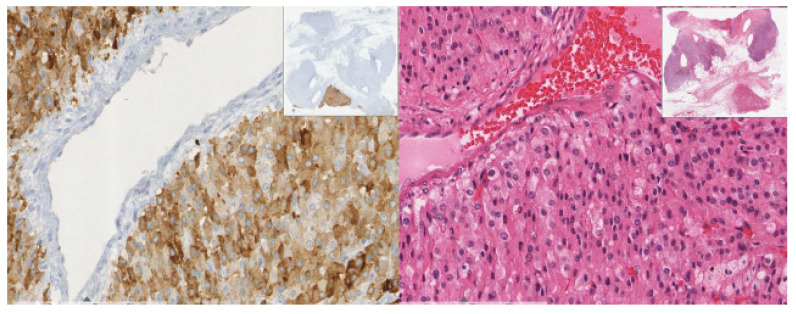
(20×): Inhibin stain: diffuse cytoplasmic staining of carcinoma component. Immunohistochemistry (IHC) for comparison.

**Figure 6:**
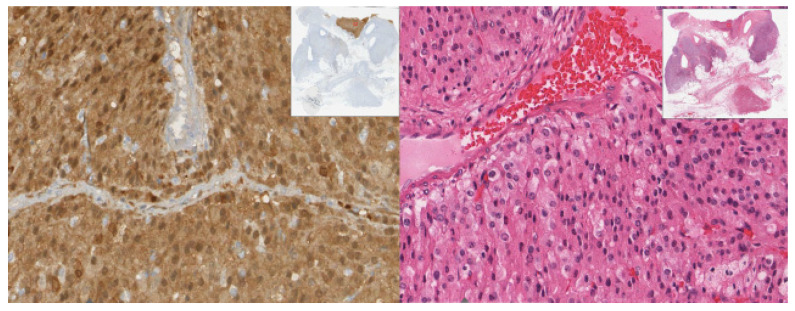
(20×): Calretinin stain: diffuse cytoplasmic and nuclear staining of carcinoma component. IHC for comparison.

**Figure 7:**
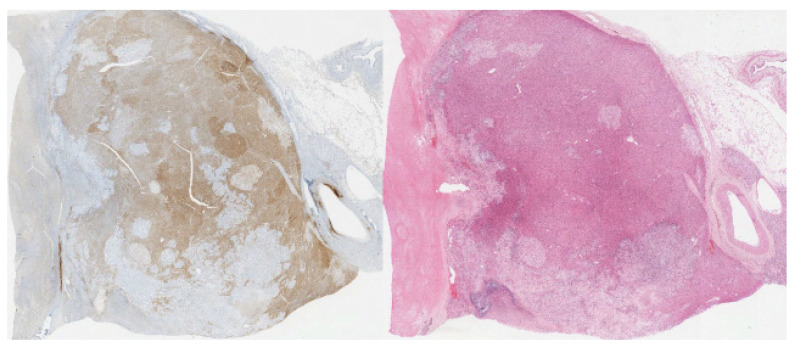
(10×): Synaptophysin stain: cytoplasmic and staining of carcinoma component. IHC for comparison.

**Figure 8:**
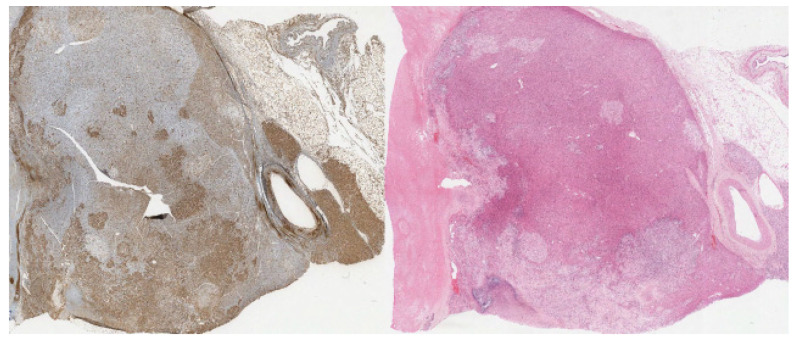
(10×): Vimentin stain: strongly positive sarcomatous component. IHC for comparison.

**Figure 9:**
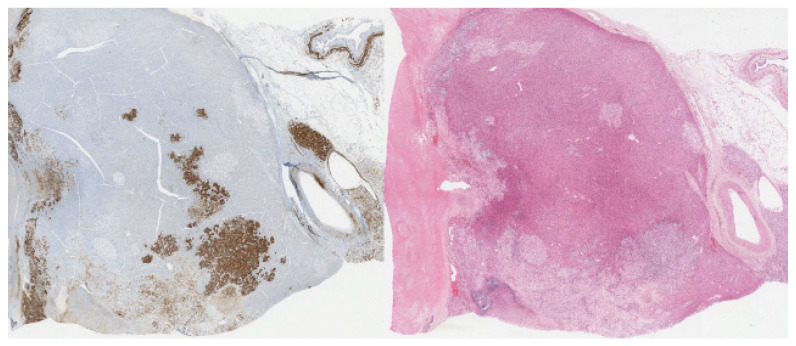
(10×): Desmin stain: strongly positive in the sarcoma component of the tumor with IHC for comparison.

The carcinoma component shows positive staining for calretinin (cytoplasmic and nuclear staining), inhibin (cytoplasmic staining), and synaptophysin (diffuse cytoplasmic and membranous staining) (Figure 5, Figure 6 and Figure 7, respectively). The sarcoma component of the tumor was stained for vimentin (cytoplasmic staining) and desmin (cytoplasmic staining) (Figure 8 and Figure 9, respectively).

The tumor cells (both carcinoma and sarcoma component) were negatively stained for HMB-45, myogenin, Pax8, and pan-keratin. The complete list of staining is described in Table 1. Based on the morphology and pattern of immunohistochemical staining, the diagnosis of the carcinosarcoma was rendered.

The surgical resection had multiple serious complications, namely bleeding and hemorrhagic shock, which necessitated transfer to the surgical intensive care unit postoperatively. The patient required massive blood transfusion, multiple vasopressor support, and continued mechanical ventilation. At this time, the patient’s family made the decision to withdraw care and pursue inpatient hospice care with comfort measures only. The patient expired shortly after.

## 3. Literature Review

Our literature review discovered 35 cases describing adrenal cortical carcinosarcomas in medical literature thus far, including this case. PubMed was utilized to conduct the literature analysis. These cases are detailed in the table below (Table 2). 

## 4. Discussion

Adrenal cortical carcinosarcoma is an extremely rare pathology. The first reported case in scientific literature was in 1987 [2]. Its defining feature is a tumor that exhibits both carcinomatous and sarcomatous differentiation [31]. The sarcomatous component can differentiate into osteosarcoma, chondrosarcoma, or rhabdomyosarcoma, or present without any recognizable differentiation with a spindle cell morphology [4]. Clinically, these malignancies can present as either nonfunctioning or functioning. Functioning tumors can show signs of cortisol excess and Cushing’s syndrome, hyperaldosteronism, or sex hormone secretion with masculinization of females and feminization of males [23].

Per an extensive literature review, we have documented 35 cases in the literature, including the case we have presented. This is the most extensive literature review of adrenal carcinosarcoma cases to date, to our knowledge. The average age at diagnosis was 55 years old with a range of 23 to 79 years old. Cases were slightly more common in females compared to males with a female-to-male ratio of 1.19. Regarding tumor location, 18 cases arose from the right adrenal gland and 12 from the left. Three cases presented with bilateral adrenal disease. The average size of the tumors was 12.4 cm across their largest dimension with average weight of 1321 g. The most common presenting complaint was pain, including abdominal, flank, back, and shoulder pain. Pain was the chief complaint in 25 (71.4%) cases. Two cases were found incidentally. Seven cases had symptoms of endocrine dysfunction, which comprised 20% of cases. The most common sarcoma histology was spindle cell, accounting for 68.6% of the cases. On presentation, 40% of patients had evidence of distant metastasis, with the liver being the most common site. All of the patients that opted for treatment underwent surgical resection, and six of these patients also received chemotherapy. Median survival from beginning of treatment until death was 4.5 months. The longest documented survival in a case was a patient that lived 2.5 years after surgery before passing [17]. Of note, there were two documented cases with patients that were alive without recurrence after surgical resection at 16 and 17 months, respectively [11,19].

Our literature review affirms the poor prognosis and aggressive behavior of adrenal carcinosarcomas [1,4]. This poor prognosis could be a result of a delay in diagnosis. Most patients possess nonfunctioning carcinosarcomas and present with symptoms caused by mass effect from the tumor with abdominal, flank, shoulder, or back pain. As such, patients on initial presentation already have a sizable disease burden. Furthermore, more than a third of patients have distant metastasis at diagnosis, and most cases will develop recurrence after surgery [1]. Another factor to consider is the difficulty in confirming the diagnosis of these malignancies. Diagnosis requires careful examination of histology. Although most of these carcinosarcomas are nonfunctioning, it can be helpful to evaluate them for hormonal dysfunction and obtain an endocrine panel with cortisol, aldosterone, and sex hormone levels when attempting to establish a diagnosis. In the cases reported above, the vast majority of adrenal cortical carcinosarcomas were visualized using computed tomography. However, imaging may be unreliable in determining the origin of these kinds of tumors. For instance, the initial hypothesis for the patient in our case was a tumor of renal origin after initial imaging was obtained.

Surgical resection is the preferred approach to obtain tissue to confirm diagnosis because there is a concern for disease spread with needle biopsy [33]. Resection is the mainstay of treatment and every documented case that pursued treatment underwent resection [24]. Due to the rarity of this malignancy, there is no standardized treatment regimen; however, adjuvant chemotherapy is typically recommended as well [24,25]. The most common chemotherapy agent utilized was mitotane, with four of the cases using this agent. The remaining two cases that underwent chemotherapy used a regimen based on etoposide and a platinum agent. Of the cases that received chemotherapy, the longest survival was noted in a patient who received treatment with mitotane; this patient was noted to be alive at 16 months after diagnosis [11]. The other five cases that underwent chemotherapy expired between 3 and 11 months, with an average survival of 6.9 months.

Carcinosarcomas are not limited to the adrenal gland. They can arise from many different organs including the uterus, ovaries, breast, prostate, lung, liver, stomach, and esophagus [34]. Perhaps the most documented are those developed from uterine tissue. Uterine carcinosarcomas, much like adrenal carcinosarcomas, have poor survival rates and high recurrence rates, and are typically treated with surgery and chemotherapy [34]. As such, there have been efforts to explore the pathogenesis of carcinosarcomas to possibly discover other treatment modalities. As the name suggests, epithelial cell adhesion molecule-1 (EpCAM) promotes cell adhesion and plays a significant role in cell differentiation, proliferation, signaling, and migration [35,36]. It is notable for being highly expressed on the cell surface of multiple malignancies and has been used as a prognostic biomarker for carcinomas and carcinosarcomas [37,38,39]. There have been treatments developed that utilize monoclonal antibodies such as solitomab and edrecolomab to target EpCAM as a means of immunotherapy for different carcinomas. These treatments have demonstrated promising antitumor activity, but they have not yet been shown to affect overall survival [37,40,41]. Nevertheless, these treatments serve as examples in the exploration of immunotherapy as a treatment modality for carcinosarcomas including adrenal carcinosarcomas.

There is also ongoing work developing new treatment modalities for adrenocortical carcinomas in general. An advanced adrenocortical carcinoma carries a dismal prognosis, with a 5-year survival rate of less than 15% [42]. Typical treatment is surgical resection, if possible, along with mitotane as adjuvant chemotherapy [43]. Therapeutics for adrenal cortical carcinoma are being considered that could target pathways such as insulin growth factor and mTOR signaling, vascular endothelial growth factor receptor (VEGF) pathway, epidermal, fibroblast, and epidermal-derived growth factor signaling, and steroidogenesis [44]. These pathways could serve as targets for treatment for adrenal carcinosarcomas as well.

Extra-gynecologic carcinosarcoma is exceedingly rare and the genomic profiling of adrenal carcinosarcoma is not readily available in the current literature. Studies performing genomic analysis in gynecologic carcinosarcomas have identified genomic aberrancies and molecular markers, which are associated with patient prognosis and can serve as potential therapeutic targets [45]. Similar studies in adrenal carcinosarcomas may identify targetable genomic alterations that can be exploited for a personalized therapy approach for the management of this aggressive malignancy.

## 5. Conclusions

Adrenal cortical carcinosarcomas are extremely rare neoplasms that are notable for exhibiting both carcinomatous and sarcomatous characteristics. There are 35 documented cases in the literature to our knowledge. Patients often present with a significant disease burden and the prognosis is usually dismal. Diagnosis can be difficult and includes complicated histology. Surgical resection is the first step in treatment and there is not a standardized treatment regimen at this time. This is the most extensive literature review of adrenal cortical carcinosarcomas to date. Continued investigations into pathogenesis are needed to explore potential treatment options. It is imperative to expand the knowledge base regarding this pathology to improve surveillance, diagnosis, treatment, and patient outcomes. Our hope is that this work can serve as a comprehensive resource and reference for other clinicians and researchers to advance research of this rare malignancy, particularly to better diagnoses and treatments.

## Figures and Tables

**Table 1:** Immunohistochemical staining in carcinomatous and sarcomatous components. All of the antibodies used in the immunology lab are pre-diluted by the manufacturing company as listed in the table.

Stain	Carcinomatous Component	Sarcomatous Component	Company	Clone
Calretinin	Positive	Negative	Ventana	SP65
HMB-45	Negative	Negative	Ventana	HMB-65
Inhibin Alpha	Positive	Negative	Ventana	R1
Synaptophysin	Positive	Negative	Ventana	SP11
Myogenin	Negative	Negative	Ventana	F5D
Vimentin	Negative	Positive	Ventana	Vim 3B4
Desmin	Negative	Positive	Ventana	DE-R-11
Pax8	Negative	Negative	Ventana	MRQ-50
Pan-keratin	Negative	Negative	Ventana	AE1/AE3/PCK 26

**Table 2:** Clinicopathologic features and outcomes of reported cases of adrenal cortical carcinosarcoma. Abbreviations: M, male; F, female; R, right; L, left; RUQ, right upper quadrant; RA, right atrium; RV, right ventricle; IVC, inferior vena cava.

Author	Age/Sex	Chief Complaint	Endocrine Dysfunction	Location	Metastasis at Presentation	Size (cm), Weight (g)	Sarcomatous Component	Treatment	Outcome
Okazumi et al., 1987 [2]	46/M	Abdominal distention, back pain	No	R	Invasion of tumor emboli to RA, RV, IVC, and retroperitoneum	14 cm, 880 g	Spindle Cell	Adrenalectomy and nephrectomy	Death at 6 months post-op
Collina et al., 1989 [5]	68/F	Abdominal discomfort	No	L	No	11 cm, Not reported	Spindle Cell	Resection	Recurrence at 2 months, Death at 6 months post-op (7 months after diagnosis)
Decorato et al., 1990 [6]	42/F	Abdominal and flank pain	No	L	No	19 cm, 1400 g	Rhabdomyosarcoma	Resection	Death at 7 months post-op
Fischler et al., 1992 [7]	29/F	Amenorrhea, fatigue, weight loss, body musculature, clitoromegaly, hirsutism	Yes	L	No	12.5 cm, 610 g	Rhabdomyosarcoma	Resection with adjuvant treatment with mitotane	Death at 8 months post-op
Barksdale et al., 1993 [8]	79/F	Severe hypertension	Yes	R	Invasion of IVC	9 cm, 199 g	Osteosarcoma, chondrosarcoma	Not reported	Not reported
Lee et al., 1997 [9]	61/M	Flank and back pain	Yes	R	Liver	12 cm, no weight reported	Spindle cell	Radical nephrectomy, right liver lobectomy	Death at 2 days post-op
Chung et al., 1998 [10]	48/F	Abdominal distention	No	R	No	Not reported	Spindle cell	Resection	Death 3 months post-op
Somda et al., 2007 [11]	58/F	Asthenia and flank pain	No	R	No	13 cm, 760 g	Leiomyosarcoma	Adrenalectomy, nephrectomy, adjuvant treatment with mitotane	Alive without recurrence after 16 months
Sturm et al., 2008 [4]	31/M	Abdominal pain	No	L	No	12 cm, 620 gm	Spindle Cell	Resection with cisplatin and etoposide	Death at 3 months post-op
Coli et al., 2010 [12]	75/F	Abdominal pain	No	L	No	15 cm, not reported	Spindle cell	Adrenalectomy and splenectomy	Death at 12 months post-op
Sasaki et. Al., 2010 [13]	45/M	Abdominal Pain, Fever, Nausea and vomiting	No	L	Left retroperitoneal invasion and bi-lobar liver metastasis	17 cm, 2974 g	Rhabdomyosarcoma	Nephrectomy, splenectomy, partial colectomy, and pancreatectomy	Death at 3 months post-op
Feng et al., 2010 [14]	72/M	Lumbar back pain	No	L	No	7.1 cm, not reported	Spindle cell	Resection	Not reported
Bertolini et al., 2011 [15]	23/F	Fatigue, decreased appetite, fixed mass in rectum	No	L	None from the primary adrenocortical carcinoma. This was a collision tumor with an adrenal metastasis of a rectal tumor	14 cm, Not reported	Osteosarcoma	adrenalectomy	Death at 14 months post-op
Thway et al., 2012 [16]	45/M	Bloating, back pain	No	L	Abdominal and retroperitoneal nodes, lung	24 cm, 6500 g	Rhabdomyosarcoma	Adrenalectomy, splenectomy, nephrectomy + combination high-dose palliative chemotherapy of vincristine, ifosfamide, doxorubicin, and etoposide, alternating with ifosfamide, carboplatin, and etoposide.	Death at 11 months post-op
Yan et al., 2012 [17]	72/M	Flank pain	No	R	Pulmonary nodules	13 cm, not reported	Spindle cell	adrenalectomy	Death at 2.5 years post-op
Kao et al., 2013 [18]	45/M	Abdominal pain, weight loss	No	R	No	15 cm, 760 g	Spindle cell	Partial nephrectomy and hepatectomy	Death at 7 months post-op
Mark et al., 2014 [19]	58/M	Flank pain	No	R	No	12 cm, 573 g	Spindle cell	Adrenalectomy and nephrectomy	No evidence of metastatic adrenal disease at 17 months
Shaikh et al., 2014 [20]	62/F	RUQ pain	No	R	Not at presentation, Para-aortic lymph nodes, 3 months after adrenalectomy	6.5 cm, 55 g	Spindle cell	adrenalectomy	Death at 4 months post-op, having declined adjuvant therapy
Wei et al., 2015 [21]	63/F	Fatigue, flank pain	No	L	No	8 cm, not reported	Spindle cell	adrenalectomy	No recurrence at one month post-op
Wanis et al., 2015 [22]	68/F	Incidental finding during follow-up of lung adenocarcinoma	No	Unspecified	No	13 cm, not reported	Spindle cell	Radical nephrectomy	Death at 223 days post-op
Wanis et al., 2015 [22]	65/M	Incidental finding during claudication work-up	No	Unspecified	No	12.8 cm, not reported	Spindle cell	Radical nephrectomy	Alive at 4 months; unknown total survival
Zhu et al., 2016 [23]	59/M	Asthenia and weight loss	No	R	Lung	5 cm, not reported	Spindle cell	adrenalectomy	Alive at 6 months post op, refused further treatment and follow up
Ishikawa et al., 2016 [3]	69/F	General malaise and hypotension	Yes	Bilateral	No	R–5.5 cm, 20 g; L–7 cm 35 g	Not reported	Resection	4 months post-op
Iyidir et al., 2016 [24]	53/F	Abdominal and flank pain, weight loss	No	Bilateral	Liver	R-9 cm, 80 g; L-8.5 cm, not reported	Spindle cell	Bilateral adrenalectomy, splenectomy, cholecystectomy, partial hepatectomy and nephrectomy	Death 1 month post-op due to pancreatic fistula development and multiorgan failure
Papathomas et al., 2016 [25]	55/M	Abdominal pain	No	L	No	16 cm, not reported	Spindle Cell	Resection	Death at 4 months from diagnosis
Papathomas et al., 2016 [25]	70/F	Abdominal pain, diarrhea	No	R	No	15 cm, not reported	Osteosarcoma, Spindle cell	Resection and mitotane	Death at 8 months from diagnosis
Papathomas et al., 2016 [25]	52/M	Abdominal pain, fatigue, malaise, weight loss	No	R	Liver	24 cm, 3020 g	Spindle Cell	Resection and mitotane	Death at 4.5 months from diagnosis
Saeger et al., 2017 [26]	53/F	Hypertension	Yes	R	Liver	13 cm, not reported	Spindle cell	Adrenalectomy and partial hepatectomy	Alive > 6 months post-op; alive at time of article but survival duration not clear
Sung et al., 2017 [27]	51/M	“Nonspecific”	No	R	Liver, spleen, lung	15 cm, not reported	Spindle cell	Resection	Death at 1.7 months
Yazir et al., 2019 [28]	52/M	Abdominal pain and distention, episodic hypertension	Yes	L	Spleen	14 cm, not reported	Not reported	Resection	Death at 1 day post-op
Sabrine et al., 2020 [29]	27/F	Flank pain	No	R	No	12 cm, 660 g	Spindle cell	Adrenalectomy	Alive at 6 months follow up without local recurrence
Rexwana et al., 2020 [30]	37/F	Facial swelling and flushing, weight gain, palpitations, RUQ abdominal pain, generalized weakness and lethargy	Yes	R	No	10 cm, not reported	Osteosarcoma	Adrenalectomy	Alive 5 months post-op, received 3 rd cycle of chemotherapy
Rachh & Nilam, 2022 [31]	78/F	Severe back pain	No	Bilateral	Bone, lymph nodes, pleura	R-4.6 cm; L-6.0 cm	Spindle cell	Not reported	Death within few months of diagnosis
Zhang et al., 2022 [32]	53/M	RUQ abdominal pain	No	R	Mediastinal Lymph Node	7.2 cm	Not reported	Surgical resection of the mass + immune therapy	6 months after diagnosis
Present Case	78/F	Fatigue and shoulder pain	No	R	No	27 cm, 3307 g	Spindle cell	Radical nephrectomy and adrenalectomy, partial hepatectomy	Death at 7 days post-op

## Data Availability

The authors declare that all data concerning this case series are provided within the manuscript.
